# KLK11 promotes the activation of mTOR and protein synthesis to facilitate cardiac hypertrophy

**DOI:** 10.1186/s12872-021-02053-y

**Published:** 2021-05-31

**Authors:** Yi Wang, Hongjuan Liao, Yueheng Wang, Jinlin Zhou, Feng Wang, Yingxin Xie, Kun Zhao, Weinian Gao

**Affiliations:** 1 Department of Ultrasound, The Second Hospital of Hebei Medical University, Shijiazhuang, China; 2 Department of Vascular Surgery, The Second Hospital of Hebei Medical University, Shijiazhuang, China; 3 Department of Critical Care Medicine, The Second Hospital of Hebei Medical University, Shijiazhuang, China

**Keywords:** KLK11, Cardiac hypertrophy, Protein synthesis, MTOR, Akt

## Abstract

**Background:**

Cardiovascular diseases have become the leading cause of death worldwide, and cardiac hypertrophy is the core mechanism underlying cardiac defect and heart failure. However, the underlying mechanisms of cardiac hypertrophy are not fully understood. Here we investigated the roles of Kallikrein 11 (KLK11) in cardiac hypertrophy.

**Methods:**

Human and mouse hypertrophic heart tissues were used to determine the expression of KLK11 with quantitative real-time PCR and western blot. Mouse cardiac hypertrophy was induced by transverse aortic constriction (TAC), and cardiomyocyte hypertrophy was induced by angiotensin II. Cardiac function was analyzed by echocardiography. The signaling pathway was analyzed by western blot. Protein synthesis was monitored by the incorporation of [^3^H]-leucine. Gene expression was analyzed by quantitative real-time PCR.

**Results:**

The mRNA and protein levels of KLK11 were upregulated in human hypertrophic hearts. We also induced cardiac hypertrophy in mice and observed the upregulation of KLK11 in hypertrophic hearts. Our in vitro experiments demonstrated that KLK11 overexpression promoted whereas KLK11 knockdown repressed cardiomyocytes hypertrophy induced by angiotensin II, as evidenced by cardiomyocyte size and the expression of hypertrophy-related fetal genes. Besides, we knocked down KLK11 expression in mouse hearts with adeno-associated virus 9. Knockdown of KLK11 in mouse hearts inhibited TAC-induced decline in fraction shortening and ejection fraction, reduced the increase in heart weight, cardiomyocyte size, and expression of hypertrophic fetal genes. We also observed that KLK11 promoted protein synthesis, the key feature of cardiomyocyte hypertrophy, by regulating the pivotal machines S6K1 and 4EBP1. Mechanism study demonstrated that KLK11 promoted the activation of AKT-mTOR signaling to promote S6K1 and 4EBP1 pathway and protein synthesis. Repression of mTOR with rapamycin blocked the effects of KLK11 on S6K1 and 4EBP1 as well as protein synthesis. Besides, rapamycin treatment blocked the roles of KLK11 in the regulation of cardiomyocyte hypertrophy.

**Conclusions:**

Our findings demonstrated that KLK11 promoted cardiomyocyte hypertrophy by activating AKT-mTOR signaling to promote protein synthesis.

## Background

During the last decades, cardiovascular diseases have become the leading cause of death worldwide [1]. The morbidity and mortality of cardiac diseases and heart failure keep growing [2]. Cardiac hypertrophy is one of the primary mechanisms that participate in the development of cardiac dysfunction and heart failure [3]. Cardiomyocytes within the heart tissues are terminally differentiated cells unable to divide to support the cardiac injury. These cardiomyocytes undergo hypertrophic growth to support the increase in demand in response to cardiac injury [4]. As thus, cardiac hypertrophy has been considered a promising target for the treatment of heart failure [3]. However, the mechanisms underlying cardiac hypertrophy remain not fully understood.

Protein synthesis is one of the critical features of cardiomyocyte hypertrophy [5]. In cardiomyocytes, protein synthesis is controlled by the mammalian target of rapamycin (mTOR) signaling pathway [6]. mTOR phosphorylated the ribosomal protein p70 S6K1 and 4EBP1 to facilitate de novo protein synthesis [7]. Within cardiomyocytes, mTOR can be regulated by various pathways. For instance, AMP-activated protein kinase (AMPK) represses mTOR and protein synthesis via regulating the phosphorylation of the TSC1/TSC2 complex, the upstream inhibitor of mTOR [8]. Besides, our current work demonstrated that Carboxypeptidase A4 promotes mTOR signaling during cardiomyocyte hypertrophy through activating PI3K-AKT signaling [9]. Furthermore, we demonstrated that 5-hydroxytryptamine receptor 2A (HTR2A) fosters the development of cardiac hypertrophy by activating PI3K-PDK1-AKT-mTOR signaling [10]. Repression of mTOR with rapamycin can inhibit protein synthesis and repress cardiac hypertrophy induced by different pathological stress [7].

Kallikreins (KLK) are a subgroup of serine proteases that have many physiological functions. Accumulating evidence suggests that many kallikreins are implicated in carcinogenesis, and some have potential as novel markers for cancer and other diseases [11]. KLK11 is a member of the human kallikrein gene family. KLK11 has significant roles in physiological and pathological processes, including cancer biology. For instance, KLK11 suppresses esophageal squamous cell carcinoma by inhibiting cellular proliferation via inhibition of the Wnt/β-catenin signaling pathway [12]. Knockdown of KLK11 reversed oxaliplatin resistance by inhibiting proliferation and activating apoptosis [13]. KLK11 also participated in colorectal adenocarcinoma [14, 15]. However, the roles of KLK11 in cardiovascular diseases, such as cardiac hypertrophy, remain to be explored. In the present work, we aimed to investigate the potential functions of KLK11 in cardiac hypertrophy and the underlying mechanisms.

## Methods

### Human samples

Five control heart samples and five hypertrophic heart samples were collected at The Second Hospital of Hebei Medical University from May 2016 to Jan 2019. The left ventricle heart tissues were frozen in liquid nitrogen and stored at − 80 °C within two hours of tissue harvesting. Each of the patients or control donors signed the written form of consent. The study was approved by the Ethics Committee of Clinical Research of Hebei Medical University. The information of the patients is shown in Table 1.

**Table 1:** Patient information in this study

Patient	Age	Gender	Diagnosis	β-adrenergic blocker	ACE inhibitor
Control-1	54	Male	Ventricular septal defect	−	−
Control-2	60	Female	Mitral valve defect	−	−
Control-3	47	Female	Ventricular septal defect	−	−
Control-4	61	Male	Ventricular septal defect	−	−
Control-5	49	Male	Mitral valve defect	−	−
Hypertrophy-1	58	Female	Hypertrophic obstructive cardiomyopathy	+	−
Hypertrophy-2	62	Male	Hypertrophic obstructive cardiomyopathy	+	+
Hypertrophy-3	65	Male	Hypertrophic obstructive cardiomyopathy	−	+
Hypertrophy-4	54	Female	Hypertrophic obstructive cardiomyopathy	−	+
Hypertrophy-5	56	Male	Hypertrophic obstructive cardiomyopathy	+	+

### Mouse model of cardiac hypertrophy

C57BL/6 mice were purchased from Charles River Laboratories (Beijing). 8–12 weeks old male C57BL/6 mice were subjected to sham or transverse aortic constriction (TAC) surgery. Four weeks later, cardiac function (fraction shortening and ejection fraction) was analyzed by echocardiography based on a protocol described previously [16]. Double-bind was applied for the echocardiography analysis. Mice were sacrificed by an overdose of isoflurane (5% isoflurane with 2 l min^−1^ oxygen) anesthesia until lack of respiration for 5 min to ensure euthanasia [17, 18], followed by heart dislocation. Heart weight, cardiomyocyte size, and gene expression were also analyzed. The animal study was approved by the Ethics Committee of Animal Research of Hebei Medical University.

### Isolation and culture of neonatal mouse cardiomyocytes

Mouse hearts were subjected to cardiomyocyte isolation based on a protocol described previously [19, 20]. The mouse cardiomyocytes were cultured in DMEM (Gibco, #C11965500BT) supplemented with 10% FBS (Gibco, #A3840001). BrdU (Sigma, #B5002) was used to repress fibroblast proliferation. Before hypertrophy induction, the cardiomyocytes were cultured in FBS-free DMEM for 24 h. Cardiomyocyte hypertrophy was induced by treatment with Angiotensin II (Ang II; MCE, #HY-13948) for 48 h in FBS-free DMEM. For KLK11 overexpression or knockdown, cardiomyocytes were infected with adenovirus or transfected with siRNA 24 h before serum starvation and Ang II treatment. Cardiomyocytes were stained with an anti-a-actinin antibody (Sigma, #A7188) for size analysis, and cell size was measured with Image J software. Fifty cardiomyocytes of each group were analyzed, and the mean cardiomyocyte size of independent experiments was used to further statistical analysis. For mTOR inhibition, 100 nM rapamycin (MCE, #HY-10219) was used.

### Gene knockdown and overexpression

The adenovirus-mediated overexpression system was applied to overexpress KLK11 in mouse cardiomyocytes. Mouse KLK11 gene ORF was cloned into adenovirus expressing vector and recombined with the construct-expressing vector based on a protocol described elsewhere [21]. The siRNA system was applied to silence the mouse KLK11 gene in cardiomyocytes. siRNA was transfected into cardiomyocytes with RNAiMAX (Invitrogen, #13778100). siCtrl sequence is 5′-GCGCGCTTTGTAGGATTCG-3′; siKLK11 sequence is 5′-GCAACATCACAGACACCAT-3′. The overexpression or knockdown efficacy was confirmed by quantitative real-time PCR and western blot.

### Adeno-associated virus 9 preparation

Adeno-associated virus 9 (AAV9) system was applied to silence mouse KLK11 gene (NM_019974.2) expression in mouse hearts. AAV9-mediated shRNA targeting control construct (AAV9-shCtrl, 5′-TTCTCCGAACGTGTCACGT-3′) or KLK11 (AAV9-shGPR39, 5′-GCAACATCACAGACACCAT-3′) were generated as described previously [22]. One week–old male C57BL/6 mice received a single intravenous injection of saline or AAV9 vecto*r *via the jugular vein, as previously described [22]. Eight weeks later, the mice received a TAC or sham surgery. The knockdown efficiency was confirmed by quantitative real-time PCR.

### Histological analysis

For histological analysis, the heart tissues were fixed in 4% paraformaldehyde (Solarbio) and then embedded in paraffin overnight. The heart tissues were cut into 5-μm slices, and hematoxylin & eosin (H&E) staining was performed to analyze the histological change with the Hematoxylin–Eosin/H&E Staining Kit (Solarbio, #G1120). The images were captured with a Nikon Microscope, and cardiomyocyte size was measured with Image J. Double-blind was applied for the histochemical analysis.

### Protein synthesis assay

[^3^H]-leucine incorporation method was used to analyze protein synthesis in cardiomyocytes, as described previously [23].

### Quantitative real-time PCR

Total RNA was extracted from heart tissues or cultured cardiomyocytes with TRIzol reagent (Invitrogen, #15596026). Then, one ug of total RNA was subjected to cDNA synthesis with the PrimeScript 1st strand cDNA Synthesis Kit (TaKaRa, #6110A). Next, quantitative real-time PCR was used to test the expression of targeted genes with the SYBR® Premix Ex Taq™ II (TaKaRa, #RR820A). Tubulin was used as a house-keeping control gene. The relative mRNA level was analyzed by the double delta Ct method. The primers used in this study are listed in Table 1.

### Western blot

Total proteins were extracted from heart tissues or cultured cardiomyocytes with RIPA reagent (Beyotime, #P0013C). Then, protein expression was analyzed by standard western blot was performed as described previously [24]. Briefly, 30 ugs of total proteins were subjected to SDS-PAGE, and then the proteins were transferred to PDVF membranes. Next, the membranes were incubated with 5% fat-free milk (TBST) for one hour, followed by TBST wash and primary antibody incubation at 4 °C overnight. The next day, the membranes were washed with TBST and incubated with accordingly secondary antibodies at room temperature for two hours. Finally, the membranes were washed with TBST, and protein expression was analyzed with an ECL kit (Millipore, #WBKLS00100). Anti-KLK11 antibody (#ab152098), anti-Tubulin antibody (#ab18207) were obtained from Abcam. Anti-AKT (#2920), anti-pAKT(#7135), anti-mTOR (#2972), anti-pmTOR (#2971), Anti-S6K1 (#9202), anti-pS6K1 (#97596), anti-4EBP1 (#9644), anti-p4EBP1(#2855) antibodies were purchased from Cell Signaling Technology. The secondary antibodies used in this study were purchased from Servicebio (#GB23301, #GB23303) (Table 2).

**Table 2:** Primers used in this study

Gene symbol	Forward primer (5′–3′)	Reverse primer (5′–3′)
Human ANP	AAGAAAGCACACCAACGCAG	GATGGTGACTTCCTCGCCTC
Human BNP	CTGATCCGGTCCATCTTCCT	TGGAAACGTCCGGGTTACAG
Human MYH7	CCAAGTTCACTCACATCCATCA	AGTGGCAATAAAAGGGGTAGC
Human KLK11	AGTGTGAGAACGCCTACCC	CCAGGAGATAATGCCTTGAAG
Human Tublin	CCAACCTGATGGGCATTGAGT	CGGCATGTAGAAGAAGGGTG
Mouse ANP	TCTTCCTCGTCTTGGCCTTT	CCAGGTGGTCTAGCAGGTTC
Mouse BNP	TGGGAGGTCACTCCTATCCT	GGCCATTTCCTCCGACTTT
Mouse MYH7	CGGACCTTGGAAGACCAGAT	GACAGCTCCCCATTCTCTGT
Mouse KLK11	ATGATTCTCCGACTCATTGCAC	TCATAACCCTTGATGATCCTCGT
Mouse Tublin	CACTGGTGTTCCGAGTGGATG	GCCAGGGTTTGACATTGACAT

### Statistical analysis

All experiments were repeated at least three times unless another statement was provided. The expression values are shown as mean ± SD. No randomization was used to allocate experimental units to control and treatment groups. The standard Student’s *t* test was applied for the analysis of the difference between the two groups. One-way ANOVA analysis followed by Tukey *post-hoc* test was performed for analysis of more than two groups. GraphPad Prism 8 software was used for all the statistical analyses, and *P* values less than 0.05 were considered significant.

## Results

### KLK11 is overexpressed in hypertrophic heart tissues.

**Fig. 1:**
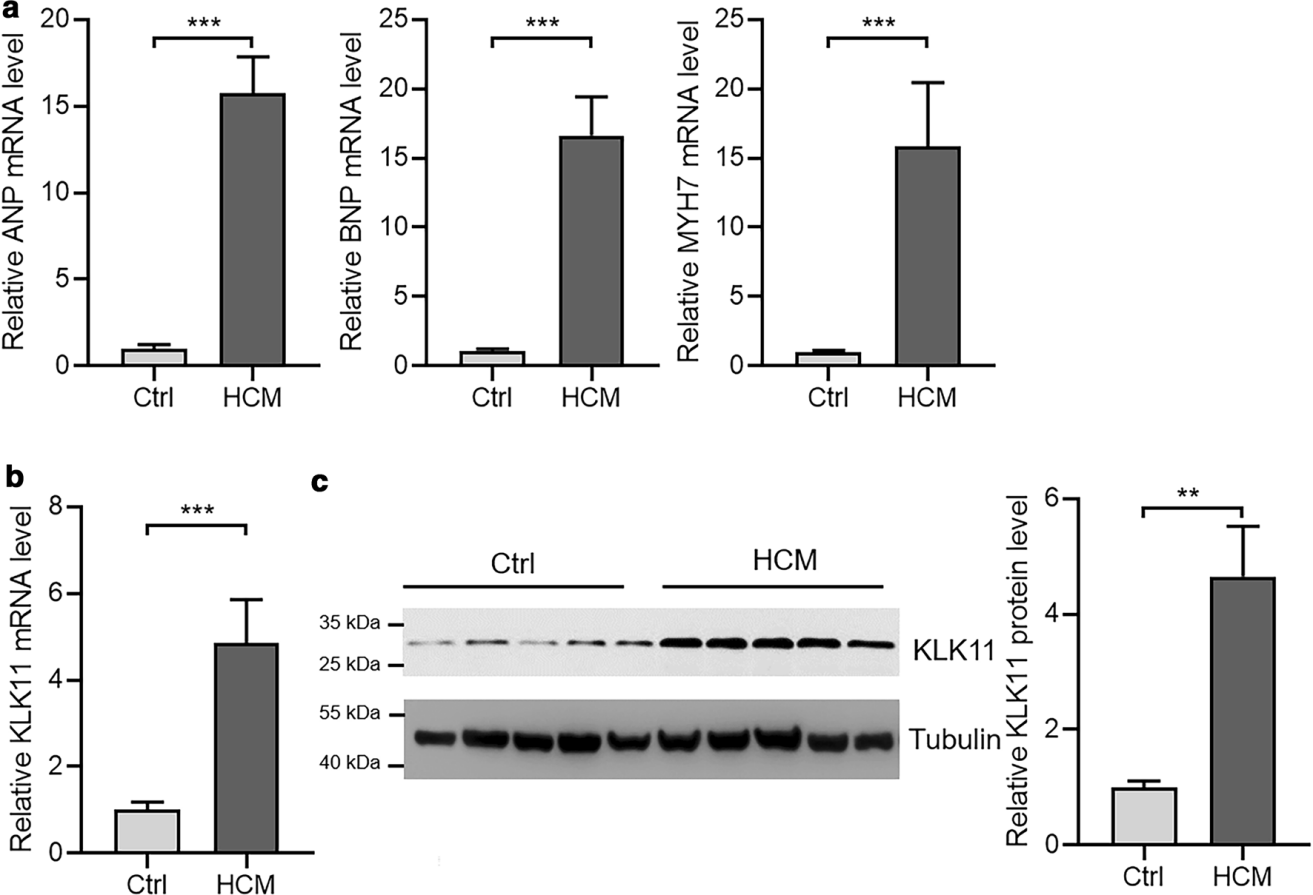
KLK11 expression is overexpressed in cardiac hypertrophy in humans. **a** Expression of mRNA of atrial natriuretic peptide (ANP), brain natriuretic peptide (BNP), and β-myosin heavy chain (MYH7) in control and hypertrophic heart tissues in humans (n = 5). ***P < 0.001 by unpaired Student’s *t* test. HCM, hypertrophic cardiomyopathy. **b** Expression of mRNA of kallikrein 11 (KLK11) in control and hypertrophic heart tissues in humans (n = 5). ***P < 0.001 by unpaired Student’s *t* test. **c** Expression of protein of KLK11 in control and hypertrophic hearts (n = 5). ***P < 0.001 by unpaired Student’s *t* test

In this study, we aimed to investigate the roles of KLK11 in cardiac hypertrophy. To this purpose, we first examined the expression of KLK11 in hypertrophic hearts of humans and mice. We collected five hypertrophic heart tissues and five control heart tissues (Table 1). The hypertrophy of human hearts was confirmed by clinical diagnosis and overexpression of hypertrophic fetal genes, ANP, BNP, and MYH7 (Fig. 1a). Then we tested the expression of KLK11 with qRT-PCR and western blot. The results showed that the expression of KLK1 mRNA and protein levels were significantly upregulated in hypertrophic human hearts (Fig. 1b, c).

**Fig. 2:**
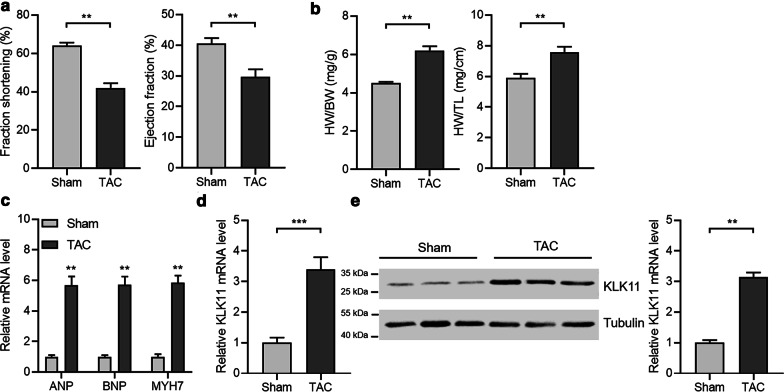
KLK11 expression is upregulated in murine hypertrophic hearts. **a** Fraction shortening and the ejection fraction of control and hypertrophic hearts in mice (n = 5). Cardiac hypertrophy in C57BL/6 mice was induced by TAC surgery for four weeks. **P < 0.01 by unpaired Student’s *t* test. **b** Heart weight of control and hypertrophic hearts in mice (n = 5). Cardiac hypertrophy in C57BL/6 mice was induced by TAC surgery for four weeks. HW, heart weight; BW, body weight; TL, tibia length. **P < 0.01 by unpaired Student’s *t* test. **c** Expression of mRNA of ANP, BNP, and MYH7 in control and hypertrophic heart tissues in mice (n = 5). Cardiac hypertrophy in C57BL/6 mice was induced by TAC surgery for four weeks. ANP, atrial natriuretic peptide; BNP, brain natriuretic peptide; MYH7, β-myosin heavy chain; **P < 0.01 *vs* Sham by unpaired Student’s *t* test. **d** Expression of mRNA of KLK11 in control and hypertrophic heart tissues in mice (n = 5). Cardiac hypertrophy in C57BL/6 mice was induced by TAC surgery for four weeks. **P < 0.001 by unpaired Student’s *t* test. **e** Expression of protein of KLK11 in control and hypertrophic heart tissues in mice (n = 3). Cardiac hypertrophy in C57BL/6 mice was induced by TAC surgery for four weeks. **P < 0.001 by unpaired Student’s *t* test

Next, we induced cardiac hypertrophy with TAC surgery in mice to test the expression of KLK11. Cardiac hypertrophy was confirmed by the decline in fraction shortening and ejection fraction, increase in heart weight, and the overexpression of hypertrophic fetal genes (Fig. 2a–c). The qRT-PCR and western blot results demonstrated that KLK11 expression was upregulated in murine hypertrophic hearts (Fig. 2d, e).

Collectively, these findings demonstrated that KLK11 was overexpressed in hypertrophic hearts of humans and mice, implicating that KLK11 may be involved in cardiac hypertrophy.

### KLK11 promotes cardiomyocyte hypertrophy in vitro

**Fig. 3:**
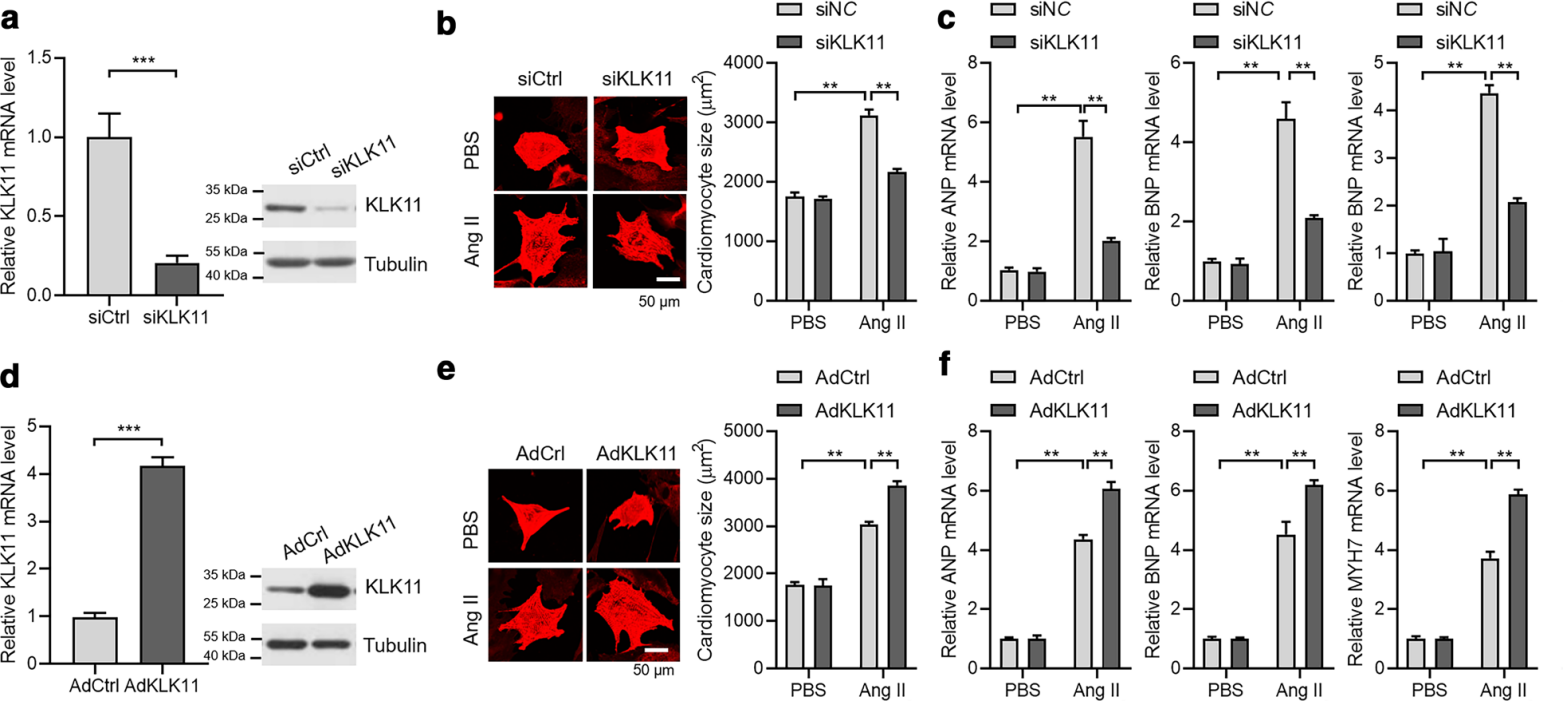
KLK11 regulates cardiac hypertrophy in vitro*.* **a** siRNA knockdown of KLK11 in cardiomyocytes (n = 3). Mouse cardiomyocytes were transfected with siCtrl or siKLK11 for 48 h. mRNA and protein levels of KLK11 were analyzed. ***P < 0.001 by unpaired Student’s *t* test. **b** KLK11 knockdown reduces Ang II-induced hypertrophic growth of cardiomyocytes (n = 3). Mouse cardiomyocytes were transfected with siCtrl or siKLK11 for 24 h, followed by Ang II (1 μM) treatment for an additional 48 h. Representative images for a-actinin staining and quantitative results are shown. **P < 0.01 by one-way ANOVA with Tukey *post-hoc* test. **c** KLK11 knockdown reduces Ang II-induced overexpression of hypertrophic genes in cardiomyocytes (n = 3). Mouse cardiomyocytes were transfected with siCtrl or siKLK11 for 24 h, followed by Ang II (1 μM) treatment for an additional 48 h. **P < 0.01 by one-way ANOVA with Tukey *post-hoc* test. **d** Adenovirus-mediated overexpression of KLK11 in cardiomyocytes (n = 3). Mouse cardiomyocytes were infected with AdCtrl or AdKLK11 for 48 h. mRNA and protein levels of KLK11 were analyzed. ***P < 0.001 by unpaired Student’s *t* test. **e** KLK11 overexpression promoted Ang II-induced hypertrophic growth of cardiomyocytes (n = 3). Mouse cardiomyocytes were infected with AdCtrl or AdKLK11 for 24 h, followed by Ang II (1 μM) treatment for an additional 48 h. Representative images for a-actinin staining and quantitative results are shown. **P < 0.01 by one-way ANOVA with Tukey *post-hoc* test. **f** KLK11 overexpression promoted Ang II-induced overexpression of hypertrophic genes in cardiomyocytes (n = 3). Mouse cardiomyocytes were infected with AdCtrl or AdKLK11 for 24 h, followed by Ang II (1 μM) treatment for an additional 48 h. **P < 0.01 by one-way ANOVA with Tukey *post-hoc* test

To test whether KLK11 promoted cardiac hypertrophy, we used an in vitro cardiomyocyte hypertrophy model. We induced cardiomyocyte hypertrophy in isolated mouse cardiomyocytes. We knocked down KLK11 with siRNA, which was confirmed by qRT-PCR and western blot (Fig. 3a). Cardiomyocyte hypertrophy was induced by angiotensin II treatment. Angiotensin II treatment increased cardiomyocyte size and promoted the expression of hypertrophy-related fetal genes. Significantly, KLK11 knockdown repressed Ang II-induced hypertrophy of cardiomyocytes as evidenced by repressed cardiomyocyte size and reduced expression of ANP, BNP, and MYH7 (Fig. 3b, c). We also overexpressed KLK11 expression with adenovirus in mouse cardiomyocytes (Fig. 3d). We observed that overexpression of KLK11 remarkedly repressed Ang II-induced increase in cardiomyocyte size and overexpression of hypertrophic fetal genes (Fig. 3e, f). Therefore, these findings demonstrated that KLK11 promoted cardiomyocyte hypertrophy.

### KLK11 promotes cardiac hypertrophy in vivo

**Fig. 4:**
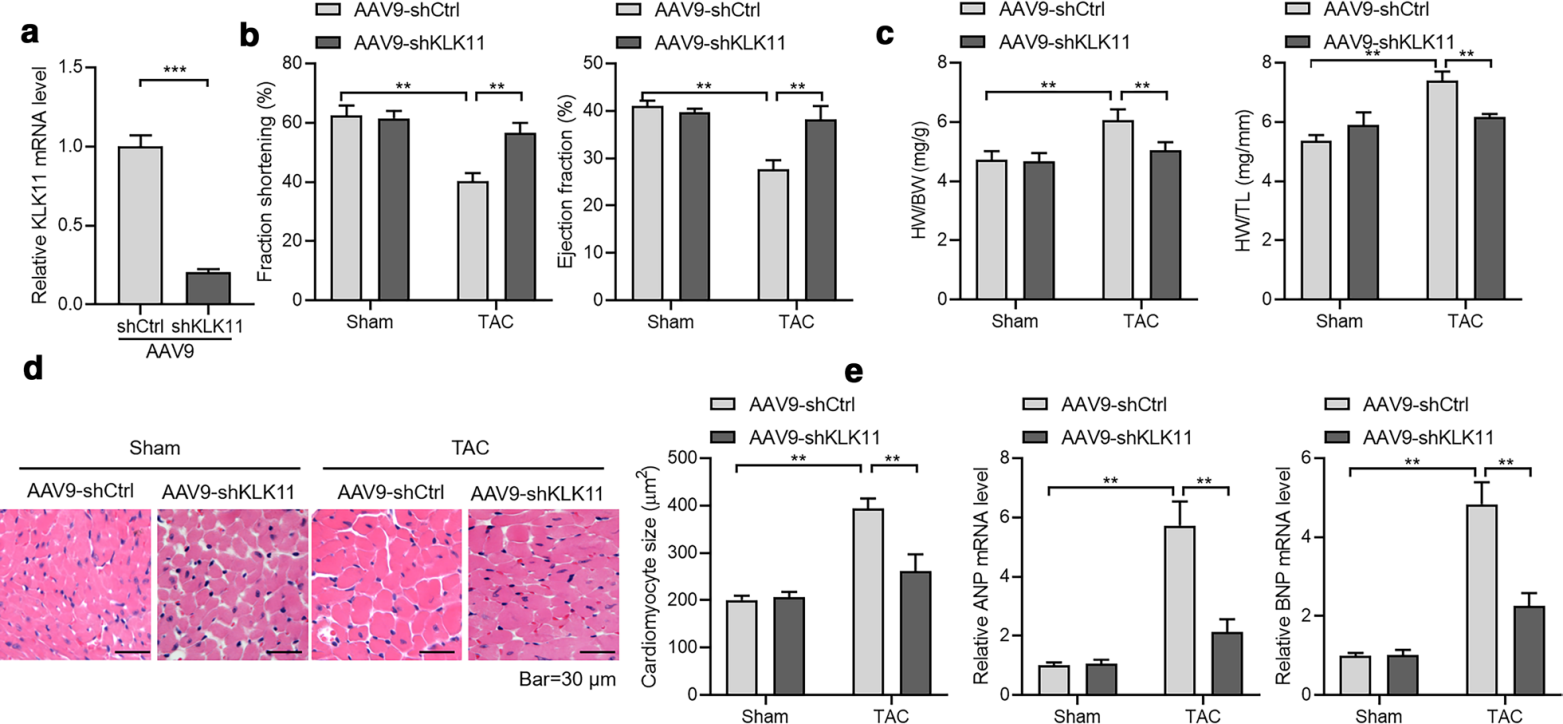
KLK11 regulates cardiac hypertrophy in vivo*.* **a** KLK11 was knocked down in murine hearts with AAV9-mediated shRNA. n = 5 in each group. One week–old male C57BL/6 mice received a single intravenous injection of saline or AAV9 vector via the jugular vein. 12 weeks later, the mice received a TAC or sham surgery. The knockdown efficiency was confirmed by quantitative real-time PCR.***P < 0.001 by unpaired Student’s *t* test. AAV9, adeno-associated virus 9. **b** KLK11 knockdown rescues TAC-induced decline in fraction shortening and ejection fraction. One week–old male C57BL/6 mice received a single intravenous injection of saline or AAV9 vector via the jugular vein. Eight weeks later, the mice received a TAC or sham surgery. n = 8 in sham groups, n = 10 in TAC groups. **P < 0.01 by one-way ANOVA with Tukey *post-hoc* test. **c** KLK11 knockdown represses TAC-induced increase in heart weight to body weight ratio (HW/BW) and heart weight to tibia length ratio (HW/TL). One week–old male C57BL/6 mice received a single intravenous injection of saline or AAV9 vector via the jugular vein. Eight weeks later, the mice received a TAC or sham surgery. n = 8 in sham groups, n = 10 in TAC groups. **P < 0.01 by one-way ANOVA with Tukey *post-hoc* test. **d** Histochemical analysis showing KLK11 knockdown represses TAC-induced increase in cardiomyocyte size. One week–old male C57BL/6 mice received a single intravenous injection of saline or AAV9 vector via the jugular vein. Eight weeks later, the mice received a TAC or sham surgery. n = 8 in sham groups, n = 10 in TAC groups.**P < 0.01 by one-way ANOVA with Tukey *post-hoc* test. **e** KLK11 knockdown represses TAC-induced increase in hypertrophic genes. One week–old male C57BL/6 mice received a single intravenous injection of saline or AAV9 vector via the jugular vein, as previously described. Eight weeks later, the mice received a TAC or sham surgery. n = 8 in sham groups, n = 10 in TAC groups. **P < 0.01 by one-way ANOVA with Tukey *post-hoc* test

To test whether KLK11 also regulates cardiac hypertrophy in vivo, we silenced KLK11 in mouse hearts with AAV9-mediated shRNA (Fig. 4a). We induced cardiac hypertrophy with TAC. We observed that the silence of KLK11 repressed TAC-induced decline in cardiac function, as monitored by fraction shortening and ejection fraction (Fig. 4b). TAC-induced increase in heart weight, cardiomyocyte size, and hypertrophic fetal gene expression was also repressed by KLK11 knockdown (Fig. 4c–e). Taken together, the data support that KLK11 plays a role in the hypertrophic response to TAC*.*

### KLK11 promotes protein synthesis

**Fig. 5:**
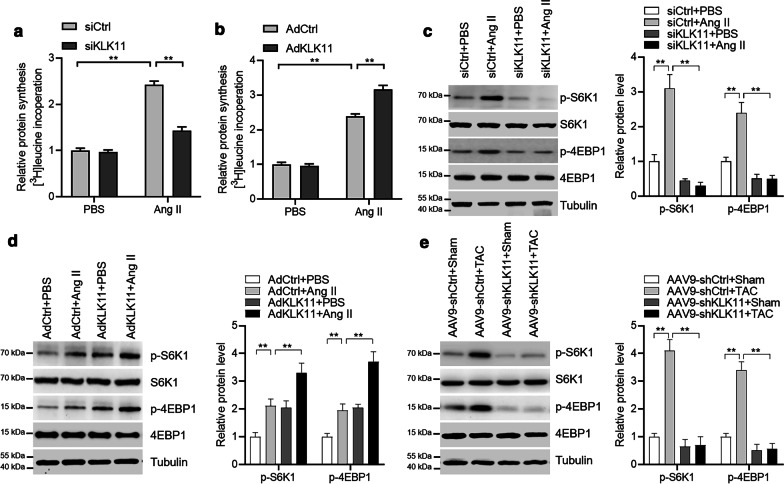
KLK11 promotes protein synthesis. **a** KLK11 knockdown represses Ang II-induced protein synthesis in cardiomyocytes (n = 3). Mouse cardiomyocytes were transfected with siRNA for 24 h, followed by protein synthesis analysis by monitoring [^3^H]-leucine incorporation in the presence of Ang II (1 μM). ***P < 0.001 by one-way ANOVA with Tukey *post-hoc* test. **b** KLK11 overexpression promotes Ang II-induced protein synthesis in cardiomyocytes (n = 3). Mouse cardiomyocytes were infected with adenovirus for 24 h, followed by protein synthesis analysis by monitoring [^3^H]-leucine incorporation in the presence of Ang II (1 μM).. ***P < 0.001 by one-way ANOVA with Tukey *post-hoc* test. **c** KLK11 knockdown represses the phosphorylation of S6K1 and 4EBP1 in cardiomyocytes (n = 3). Mouse cardiomyocytes were transfected with siRNA for 24 h, followed by Ang II (1 μM) treatment for an additional 24 h. Representative western blots and quantitative results are shown. **P < 0.001 by one-way ANOVA with Tukey *post-hoc* test. **d** KLK11 overexpression promotes phosphorylation of S6K1 and 4EBP1 in mouse cardiomyocytes (n = 3). Mouse cardiomyocytes were infected with adenovirus for 24 h, followed by Ang II (1 μM) treatment for an additional 24 h. Representative western blots and quantitative results are shown. **P < 0.01 by one-way ANOVA with Tukey *post-hoc* test. **e** KLK11 knockdown represses the phosphorylation of S6K1 and 4EBP1 in vivo (n = 3). One week–old male C57BL/6 mice received a single intravenous injection of saline or AAV9 vector via the jugular vein. Eight weeks later, the mice received a TAC or sham surgery. Heart tissues from hypertrophic hearts with/without KLK11 knockdown were analyzed. Representative western blots and quantitative results are shown. **P < 0.01 by one-way ANOVA with Tukey *post-hoc* test

Protein synthesis is one of the critical features of hypertrophic growth [7]. As thus, we tested whether KLK11 regulated protein synthesis. Indeed, we observed that KLK11 overexpression promoted whereas KLK11 silence repressed Ang II-induced increase in protein synthesis in mouse cardiomyocytes (Fig. 5a, b). Protein synthesis was controlled by S6K1 and 4EBP1 [25–27]. We observed that KLK11 overexpression activated, whereas KLK11 knockdown repressed the phosphorylation of S6K1 and 4EBP1 in mouse cardiomyocytes (Fig. 5c, d). Besides, we also analyzed the effects of KLK11 on S6K1 and 4EBP1 in vivo. The results showed that KLK11 silence repressed S6K1 and 4EBP1 phosphorylation in hypertrophic mouse hearts (Fig. 5e). Collectively, these results demonstrated that KLK11 activated S6K1 and 4EBP1 to promote protein synthesis and cardiomyocyte hypertrophy.

### KLK11 regulates protein synthesis and cardiomyocyte hypertrophy via mTOR

**Fig. 6:**
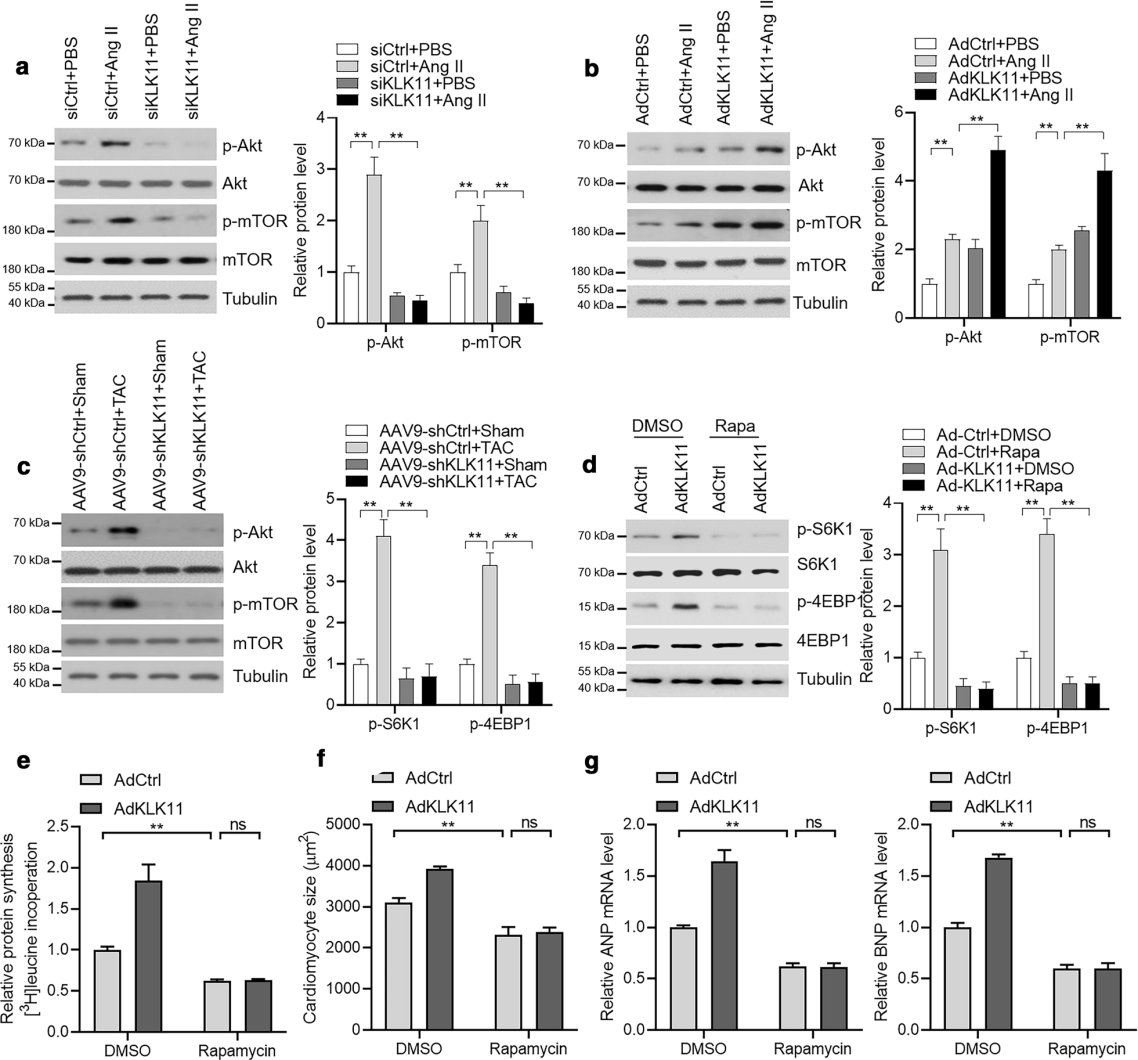
KLK11 regulates mTOR signaling to target cardiac hypertrophy. **a** KLK11 knockdown represses the phosphorylation of Akt-mTOR (n = 3). Mouse cardiomyocytes were transfected with siRNA for 24 h, followed by Ang II (1 μM) treatment for an additional 24 h. Representative western blots and quantitative results are shown. **P < 0.01 by one-way ANOVA with Tukey *post-hoc* test. **b** KLK11 overexpression promotes phosphorylation of Akt-mTOR (n = 3). Mouse cardiomyocytes were infected with adenovirus for 24 h, followed by Ang II (1 μM) treatment for an additional 24 h. Representative western blots and quantitative results are shown. **P < 0.01 by one-way ANOVA with Tukey *post-hoc* test. **c** KLK11 knockdown represses the phosphorylation of Akt-mTOR in vivo (n = 3). One week–old male C57BL/6 mice received a single intravenous injection of saline or AAV9 vector via the jugular vein. Eight weeks later, the mice received a TAC or sham surgery. Heart tissues from hypertrophic hearts with/without KLK11 knockdown were analyzed. Representative western blots and quantitative results are shown. **P < 0.01 by one-way ANOVA with Tukey *post-hoc* test. **d** Rapamycin represses KLK11-mediated phosphorylation of S6K1 and 4EBP1 (n = 3). Mouse cardiomyocytes were infected with adenovirus for 24 h, followed by Ang II (1 μM) treatment in the presence of rapamycin (100 nM) for an additional 24 h. Representative western blots and quantitative results are shown. **P < 0.01 by one-way ANOVA with Tukey *post-hoc* test. **e** Rapamycin represses KLK11-mediated protein synthesis (n = 3). Mouse cardiomyocytes were infected with adenovirus for 24 h, followed by protein synthesis by monitoring [^3^H]-leucine incorporation in the presence of Ang II (1 μM) and rapamycin (100 nM). ***P < 0.001 by one-way ANOVA with Tukey *post-hoc* test. **f** Rapamycin represses KLK11-mediated cardiomyocyte hypertrophic growth (n = 3). Mouse cardiomyocytes were infected with adenovirus for 24 h, followed by Ang II (1 μM) treatment in the presence of rapamycin (100 nM) for an additional 48 h. ***P < 0.001 by one-way ANOVA with Tukey *post-hoc* test. **g** Rapamycin represses KLK11-mediated overexpression of hypertrophic genes (n = 3). Mouse cardiomyocytes were infected with adenovirus for 24 h, followed by Ang II (1 μM) treatment in the presence of rapamycin (100 nM) for an additional 48 h. ***P < 0.001 by one-way ANOVA with Tukey *post-hoc* test

Finally, we explored the mechanism by which KLK11 promoted protein synthesis and cardiomyocyte hypertrophy. AKT-mTOR is a critical upstream regulator for SK61, 4EBP1, and subsequently, protein synthesis [7]. We tested whether AKT1-mTOR signaling was involved in the KLK11 function in protein synthesis. We observed that KLK11 knockdown repressed, whereas KLK11 overexpression activated the AKT-mTOR signaling pathway in mouse cardiomyocytes (Fig. 6a, b). Besides, KLK11 effects on the AKT-mTOR signaling pathway was also observed in hypertrophic mouse hearts (Fig. 6c). To test whether mTOR was accounted for KLK11 effects on S6K1, 4EBP1, and protein synthesis, we inhibited mTOR with rapamycin. mTOR signaling can be induced within 24 h, and the activation can sustain later time points during these incubations. By contrast, rapamycin is an inhibitor of mTOR and can repress mTOR phosphorylation within hours [26, 27]. Inhibition of mTOR repressed KLK11-mediated activation of S6K1 and 4EBP1 and inhibited KLK11-increased protein synthesis in mouse cardiomyocytes (Fig. 6d, e). Furthermore, we also found that rapamycin blocked the effects of KLK11 overexpression on cardiomyocyte size and expression of hypertrophy-associated fetal genes (Fig. 6f, g). Collectively, these findings demonstrated that KLK11 promoted AKT-mTOR signaling to activate protein synthesis and promote cardiac hypertrophy.

## Discussion

In this work, we identified KLK11 as a regulator of cardiac hypertrophy. The expression of KLK11 was upregulated in the hypertrophic hearts of humans and mice. Our in vitro loss-of-function and gain-of-function as well as in vivo loss-of-function experiments demonstrated that KLK11 promoted cardiomyocyte hypertrophy in vitro and in vivo. In cardiomyocytes, KLK11 activated S6K1 and 4EBP1 to promote protein synthesis. Mechanism study showed that KLK11 activated AKT-mTOR signaling to promote the activation of S6K1 and 4EBP1 and protein synthesis. Inhibition of mTOR with rapamycin blocked the effects of KLK11 on protein synthesis and cardiomyocyte hypertrophy.

KLK11 is a member of the kallikrein family [28]. Accumulating evidence showed that KLK11 critically participated in the development and drug resistance of human cancer. For instance, KLK11 mRNA expression predicts poor disease-free and overall survival in patients with colorectal adenocarcinoma [14]. In human colorectal cancer cells, KLK11 knockdown inhibited cell proliferation and increases oxaliplatin sensitivity [13, 15]. In addition to cancer biology, KLK11 also participated in other pathological processes. For example, elevated immunoglobulin to tissue KLK11 was observed in patients with Sjögren syndrome [29]. Although the roles of KLK11 have been identified in cancer biology and Sjögren syndrome [13, 15, 29], the roles of KLK11 in cardiovascular diseases are largely unknown.

Here we reported the roles of KLK11 in cardiac hypertrophy. Using human hypertrophic heart tissues, we demonstrated the overexpression of KLK11 in cardiac hypertrophy. Besides, we induced cardiac hypertrophy in mice with TAC surgery. In the mouse model of cardiac hypertrophy, we also observed the overexpression of KLK11. These results implicated the potential involvement of KLK11 in cardiac hypertrophy, which may be a conserved mechanism underlying cardiac hypertrophy across species. However, the mechanism by which KLK11 was activated in hypertrophic hearts remains unknown. We analyzed the promoter of KLK11 and identified multiple NFATC3 binding sites (data not shown). NFATC3 is a transcriptional factor for cardiac hypertrophy [1, 5]. The overexpression of KLK11 in hypertrophic hearts may be due to NFATC3 activation. Further studies are needed to test this hypothesis.

By using in vitro model of cardiomyocyte hypertrophy, we demonstrated that KLK11 overexpression promoted Ang II-induced hypertrophic growth whereas KLK11 knockdown repressed hypertrophic growth of cardiomyocytes. Besides, we knocked down KLK11 in mouse hearts and observed that KLK11 loss suppressed TAC-induced decrease in heart function (fraction shortening and ejection fraction) and increase in heart weight, cardiomyocyte size, as well as overexpression of fetal genes associated with cardiac hypertrophy. To our knowledge, this was the first report about the roles of KLK11 in cardiovascular disease. A previous report showed that KLK8 was expressed in the myocardium and induced cardiac hypertrophy [30]. As thus, the KLK family may have a similar function in cardiac hypertrophy. However, the roles of other KLK family members in cardiovascular diseases are far from understood.

Protein synthesis is the core mechanism underlying cardiomyocyte hypertrophy [5]. We observed that KLK11 promotes protein synthesis during cardiomyocyte hypertrophy by activating the key regulators, S6K1 and 4EBP1. Mechanism study identified AKT-mTOR as a target of KLK11 in hypertrophic cardiomyocytes and hearts. mTOR is the pivotal upstream activator of S6K1 and 4EBP1 and contributed significantly to cardiac hypertrophy [7]. Inhibition of mTOR with rapamycin reduced protein synthesis, expression of hypertrophy-associated fetal genes such as ANP and BNP, and subsequently repressed cardiac hypertrophy in vivo and cardiomyocyte hypertrophy in vitro [7, 26, 27]. We observed that inhibition of mTOR with rapamycin blocked the effects of KLK11 on S6K1, 4EBP1, and repressed KLK11-mediated promotion of protein synthesis and cardiomyocyte hypertrophy. Previous studies have identified several downstream effectors of KLK11, including Wnt, IGFBP3[12, 28]. Here we identified the AKT-mTOR signaling pathway as a novel downstream effector of KLK11. Previous studies showed that KLK11 promoted cell proliferation [12, 13], which may also be contributed by KLK11-mediated activation of mTOR and protein synthesis. The role of KLK11 in AKT-mTOR and protein synthesis support a novel involvement of KLK11 in nutrient sensing and intracellular mechanism, as thus KLK11 may participate in diverse physiological and pathological progress. Although some studies have identified the roles of KLF11 in cancer biology [12–15, 30], the functions of KLK11 in other metabolism-related diseases are still covered.

## Conclusion

In the present work, we demonstrated that KLK11 promoted the development of cardiac hypertrophy via activating AKT-mTOR signaling and protein synthesis. Therefore, KLK11 may serve as a target for the treatment of cardiac hypertrophy.

## Data Availability

All data generated or analyzed during this study are included in this published article [and its supplementary information files].

## Abbreviations

HCM: Hypertrophic cardiomyopathy
ANP: Atrial natriuretic peptide
BNP: Brain natriuretic peptide
MYH7: β-Myosin heavy chain
TAC: Transverse aortic constriction
mTOR: Mammalian target of rapamycin
